# Association of Combined Marijuana and Opioid Use with Major Depressive Disorder Among Adults with Chronic Conditions in the United States

**DOI:** 10.1177/29768357261423812

**Published:** 2026-02-23

**Authors:** Tiffany Graham, R. Constance Wiener, Sophie Mitra, Hao Wang, Chan Shen, Laskhmi Pedaprolu, Usha Sambamoorthi

**Affiliations:** 1 Pharmacotherapy Department, College of Pharmacy, University of North Texas Health Sciences Center, Fort Worth, TX, USA; 2 Department of Dental Public Health and Professional Practice, School of Dentistry, West Virginia University, Morgantown, WV, USA; 3 Department of Economics, Fordham University, Bronx, NY, USA; 4 Department of Emergency Medicine, Integrative Emergency Services, JPS Health Network, Fort Worth, TX, USA; 5 Department of Surgery, Penn State College of Medicine, Pennsylvania State University, Hershey, PA, USA

**Keywords:** marijuana, opioids, chronic conditions, co-use of marijuana and opioids, major depressive disorder (MDD), NSDUH

## Abstract

**Background:**

Individuals with chronic conditions often use opioids for pain and the broadening legalization of marijuana and its potential analgesic effects may prompt some individuals to use marijuana as an additional option. Both substances have been individually linked to adverse effects on major depressive disorder (MDD), but little is known about their co-use. This study examined the association between co-use and MDD among US adults with chronic conditions.

**Methods:**

This cross-sectional analysis used 2022 National Survey of Drug Use and Health data from 35 585 adults older than 18 years with chronic conditions. Participants were categorized into 4 groups: (1) co-use of marijuana and opioids, (2) opioid only, (3) marijuana only, and (4) neither in the past year. MDD was defined using DSM-5 criteria (⩾2 weeks of depressed mood or anhedonia with ⩾5 symptoms). Associations between substance use and MDD were assessed with Rao-Scott chi-square tests and multivariable logistic regression, adjusting for demographic, behavioral, clinical, and social determinants of health as well as COVID-19-related mental health and past-year benzodiazepine use.

**Results:**

Overall, 8.5% reported co-use; 26.1% reported opioid use and no marijuana use; and 10.9% reported marijuana use and no opioid use. Past year MDD was highest among those with co-use (20.8%) followed by marijuana only (18.4%); opioid only (9.3%); and neither (5.6%). In fully adjusted logistic regression, co-use had the strongest association with past year MDD (AOR = 1.92, 95% CI = 1.45, 2.50) followed by marijuana only (AOR = 1.72, 95% CI = 1.31, 2.26) and opioid only (AOR = 1.44, 95% CI = 1.14, 1.82) compared to the neither opioids nor marijuana group.

**Conclusion:**

Co-use of opioids and marijuana is not uncommon among adults with chronic conditions and is associated with higher odds of MDD. These findings underscore the importance of monitoring substance use in people with chronic conditions, especially to detect co-use that may worsen mental health.

## Introduction

Existing research demonstrates that chronic pain frequently co-occurs with chronic physical illnesses, with prevalence rates ranging from 26% to nearly 50%, depending on the condition.^1^ For example, pain affects approximately 46% of adults with diabetes, 41% with heart disease, 33% with asthma, and almost half of those with chronic obstructive pulmonary disease (COPD).^1^ Managing pain in chronic conditions involves the use of analgesics medications. When conventional treatments such as nonsteroidal anti-inflammatory drugs (NSAIDs), topical agents, or corticosteroids do not provide adequate relief, opioids are often prescribed as part of a pain management plan. For example, in 2019, 22.1% of U.S. adults with chronic pain reported using prescription opioids within 3 months.^2^

However, the long-term benefits of opioids for chronic pain management are limited, and national prescribing guidelines have shifted to reflect growing safety concerns.^3^ Therefore, in recent years, marijuana has also emerged as an alternative or adjunctive therapy for pain management.^4^ In 2022, 31% of adults reported using marijuana to manage their pain.^5^ However, evidence regarding marijuana’s effectiveness is mixed: a large prospective study found no significant pain-relieving benefit,^6^ whereas a systematic review of 15 randomized clinical trials reported modest benefits for certain chronic conditions.^7^

Despite mixed evidence, national efforts to curb opioid prescribing may have accelerated the use of marijuana as a substitute or companion therapy.^8,9^ In the US, states that have legalized marijuana use report lower opioid prescribing rates, suggesting that marijuana may partially replace opioids for pain management.^10,11^ Some adults report using marijuana to reduce opioid consumption,^12-14^ enhance analgesic effects when combined with low-dose opioids,^15^ or achieve better overall pain control.^16,17^

As a result, co-use of opioids and marijuana may be increasingly common. For example, among U.S. veterans prescribed opioids, 9.7% reported marijuana co-use in 2018,^18^ and among individuals with chronic pain treated with opioids, 8.6% reported co-use.^19^ Among pregnant women treated for opioid use disorder, co-use prevalence ranged from 24%^20^ to 45%.^21^ Given these patterns of co-use, it is critical to consider the broader mental health implications of opioid and marijuana use. Separate lines of research have shown a strong link between opioid use and depression,^22^ potentially mediated by emotion dysregulation^23^ or genetic vulnerability.^24^ Likewise, marijuana use has been associated with an increased risk of depressive disorders.^25,26^ For example, a systematic review of 14 longitudinal studies concluded that frequent and heavy consumption of marijuana could lead to increased risk of incident depressive disorders,^25^ potentially due to structural and functional changes in emotion-regulating brain regions such as the amygdala and prefrontal cortex.^27^ It is plausible that co-use of substances may compound this risk. For example, in comparison to those who used only opioids, individuals who co-used opioids and cannabis exhibited heightened anxiety and depression symptoms.^28^ In a study of participants from 2 large pain centers in Israel, those with co-use had higher adjusted odds of depression (aOR = 3.34, 95% CI = 1.52, 7.34) compared to those with only marijuana use.^19^

Adults with chronic physical conditions represent an important population in which to understand these patterns, given their higher likelihood of experiencing pain and using multiple substances for pain relief. However, research on the association of co-use with depression, among adults with chronic conditions in the US remains limited, highlighting the need for further investigation in this area. Therefore, the aim of this study is to investigate the association between the co-use of opioids and marijuana and MDD among adults with chronic conditions in the US. Based on existing evidence linking substance co-use to adverse mental health outcomes, we hypothesized that individuals reporting co-use would have higher odds of MDD compared to those reporting no use.

## Methods

### Study Design

We used a cross-sectional study design. The data source was the 2022 National Survey of Drug Use and Health (NSDUH, 2022). NSDUH is an annual survey for the collection of self-reported data on tobacco, alcohol and drug use, and health status. NSDUH has a nationally representative sample of non-institutionalized individuals in the US. Their 2022 weighted sample covered approximately 224 million adults representing the US non-institutionalized population. We focused on individuals aged 18 or older (n = 15 527) with chronic conditions. In NSDUH the respondents were provided with a list of chronic conditions and were asked “*Below is a list of health conditions that you may have had during your lifetime. Please read the list and type in the numbers of all of the conditions that a doctor or other health care professional has ever told you that you had.*” The following conditions were listed: asthma, cancer, kidney disease, cirrhosis of the liver, chronic obstructive pulmonary disease, diabetes, heart disease, hypertension, hepatitis B or C, and HIV or AIDS. Adults who have never had any of these conditions were classified as a “No chronic condition” group. Thus, our analytical sample consisted of adults aged 18 or older only with the chronic conditions listed above.

## Measures

### Dependent Variable: Major Depressive Disorder (Yes/No)

NSDUH defined MDD based on Diagnostic and Statistical Manual of Mental Disorders (DSM-5) criteria.^29,30^ Based on DSM-5 criteria, those who felt depressed or lost interest or pleasure in daily activities for ⩾2 weeks in the past year and those experiencing ⩾5 of 9 symptoms during the same 2-week period were classified as MDD.

### Key Independent Variable: Marijuana and Opioid Use Categories

Opioid use in the NSDUH was identified if the participant used opioids as a prescription medication (as directed by a doctor) or used in any way not directed by a doctor in terms of amount, duration, other during the previous year (yes, no). Separation of prescription use, and opioid use not directed by a doctor was not available in the NSDUH, 2022. Participants were also asked if they had any marijuana product use during the past 12 months (yes, no). We combined opioid and marijuana use to create the following 4 mutually exclusive groups of past year use: (1) co-use of opioids and marijuana; (2) opioid only; (3) marijuana only; and (4) no use of opioids/marijuana. Our marijuana measure reflects any past-year use regardless of purpose or source.

### Other Independent Variables

As MDD is affected by many factors, we also included other explanatory variables such as age, sex, race and ethnicity education, poverty status, marital status, and insurance coverage. We also included health-related variables (multimorbidity) and lifestyle factors such as smoking, alcohol use, and obesity (body mass index). As COVID-19 has been shown to affect mental health,^31^ NSDUH has a question on whether COVID-19 negatively affected mental health (not at all, a little or some, quite a bit or lot). Because the COVID-19 pandemic had a profound and lasting impact on mental health, we included this NSDUH variable on self-reported COVID-19-related mental health effects as a covariate. Multiple studies have documented that COVID-19-related stress, isolation, and socioeconomic disruptions substantially increased the prevalence and severity of depressive and anxiety symptoms in the general population, particularly among adults with chronic conditions who were already vulnerable to psychological distress.^32-34^ Including this variable allowed us to account for pandemic-related mental health changes that might confound the relationship between opioid and marijuana use and major depressive disorder. We included benzodiazepine as it is used to treat MDD.^35^ As MDD is affected by many factors, we also included other explanatory variables such as demographics (age, sex, race, and ethnicity), social determinants of health (SDOH) including education, poverty status, marital status, and insurance coverage and lifestyle factors (smoking, alcohol use, and obesity). Moreover, we included multimorbidity, defined as having 2 or more chronic conditions. Detailed chronic conditions percentages for our study sample are provided in the Appendix.

### Statistical Analyses

Rao-Scott Chi-square tests that accounted for clustering, stratification and weights were used to analyze the unadjusted association of opioid and marijuana use categories with MDD. Nested multivariable logistic regressions were used to adjust for other explanatory variables that may be associated with MDD. We built 4 nested models. The first model included only opioid and marijuana use categories; model 2 additionally included sex, age, and race and ethnicity. Model 3 furthermore included SDOH. The fully adjusted logistic regression model (Model 4) incorporated age, sex, race, and ethnicity, SDOH, benzodiazepine use, multimorbidity, body mass index, smoking and alcohol use and negative mental health effects due to COVID-19. All analyses were conducted in SAS 9.4 using survey procedures (SAS Institute, Cary, NC).

## Results

In this study of adults with chronic conditions, 52.2% were female, 66.7% were non-Hispanic White, and 12.2% were non-Hispanic Black. 37.2% had multimorbidity, defined as the concurrent presence of 2 or more chronic conditions (Table 1). There were 8.5% reporting co-use of opioids and marijuana; 26.1% reported opioid use only; 10.9% reported marijuana use only; and over half (54.5%) reported no opioid or marijuana use within the past year.

**Table 1.** Description of Selected Characteristics Among Adults (⩾18 years) with Chronic Conditions National Survey Drug Use and Health (NSDUH), 2022.

Group	N	Wt N	Wt%
All	15 527	109 066 757	100.0
Marijuana and opioid use past 12 mo			
Co-use	1527	9 278 904	8.5
Opioids only	3541	28 489 614	26.1
Marijuana only	2321	11 838 970	10.9
Neither	8138	59 459 269	54.5
Age in years			
18-23	2017	5 152 378	4.7
24-34	2957	11 071 205	10.2
35-49	4072	20 032 975	18.4
50-64	2791	32 542 411	29.8
65 or older	3690	40 267 787	36.9
Sex			
Female	8883	56 922 347	52.2
Male	6644	52 144 410	47.8
Race and ethnicity			
Non-Hispanic White	10 254	72 780 520	66.7
Non-Hispanic Black	1779	13 273 996	12.2
Hispanic	1996	14 629 174	13.4
Non-Hispanic Asian	645	5 298 188	4.9
Other	853	3 084 878	2.8
Marital status			
Married	7362	56 539 047	51.8
Widowed	927	10 548 044	9.7
Divorced/separated	2102	18 907 946	17.3
Never married	5136	23 071 721	21.2
Education			
Less than high school	1443	9 986 593	9.2
High school	3734	29 960 699	27.5
Some college/associate	4870	34 042 449	31.2
College	5480	35 077 015	32.2
Poverty level			
<100% FPL	2409	14 413 473	13.2
100%-200%	3054	21 308 143	19.5
>200% FPL	10 060	73 344 236	67.2
Insurance coverage			
Private insurance	9318	64 196 668	58.9
Public insurance	5286	39 479 881	36.2
No insurance	923	5 390 208	4.9
Metropolitan status			
Large metro	6778	57 462 368	52.7
Small metro	6235	34 884 729	32.0
Non-metro	2514	16 719 660	15.3
Multimorbidity			
Yes	4866	40 623 567	37.2
No	10 661	68 443 190	62.8
Body Mass Index			
Underweight/normal	4064	26 704 254	24.5
Overweight	4414	33 461 012	30.7
Obese	6723	46 540 849	42.7
Smoking status			
Current smoker	2180	15 699 430	14.4
Former smoker	3545	29 028 915	26.6
Never smoked	9787	64 088 901	58.8
Alcohol use			
Heavy drinker	742	4 629 331	4.2
Moderate drinker	7177	48 128 645	44.1
Past month use only	5306	39 727 982	36.4
Never	2065	15 315 323	14.0
Benzodiazepine use within the year			
Yes	2002	14,143770	13.0
No	13 525	94 922 987	87.0
COVID and mental health			
No effect	5044	40 790 657	37.4
Little effect	7731	52 902 933	48.5
Quite a bit/a lot	2539	14 363 358	13.2

Abbreviation: Wt, weighted.

Based on 15 527 adult participants aged 18 years or older with chronic conditions from the 2022 National Survey on Drug Use and Health. Missing categories of poverty status, alcohol use, smoking status, body mass index, and COVID variables are not presented.

Table 2 provides the frequencies and weighted percentages of opioid and marijuana use categories by individual characteristics. Females had a higher prevalence of opioid-only use compared to males (28.9% vs 23.1%; *P* < .001). Males had a higher prevalence of marijuana-only use compared to females (12.4% vs 9.4%; *P* < .001). Considering age, the highest prevalence of co-use of opioids and marijuana was among adults aged 25 to 34 years (15.0%), whereas adults aged ⩾ 65 years had the lowest prevalence (3.7%; *P* < .001). In terms of race/ethnicity, non-Hispanic White and non-Hispanic Black individuals had the highest opioid-only use (27.5%), while Asians had the lowest (19.3%; *P* < .001). Rates of co-use of opioids and marijuana were similar among non-Hispanic Black (8.1%) and non-Hispanic White individuals (8.8%), and lower among Hispanics (7.5%) and Asians (2.5%).

**Table 2.** Description of Selected Characteristics by Marijuana & Opioid Use Among Adults (>18 years) with Chronic Conditions National Survey of Drug Use and Health, 2022.

	Co-Use		Opioid only		Marijuana only		Neither		*P*-value
Group	N	Wt%	N	Wt%	N	Wt%	N	Wt%	
All	1527	8.5%	3541	26.1	2321	10.9	8138	54.5	
Sex									<.001
Female	928	7.8	2230	28.9	1213	9.4	4512	53.9	
Male	599	9.3	1311	23.1	1108	12.4	3626	55.2	
Age in years									<.001
18-23	238	11	240	10.7	584	31.9	955	46.7	
24-34	411	15	450	15.8	750	24.5	1346	44.8	
35-49	508	14	983	23.8	591	14.6	1990	47.9	
50-64	227	8.8	797	28.8	258	9.5	1509	52.9	
65+	143	3.7	1071	29.9	138	3.7	2338	62.7	
Race and ethnicity									<.001
Non-Hispanic White	1007	8.8	2391	27.5	1467	10.6	5389	53	
Non-Hispanic Black	168	8.1	487	27.5	270	11.8	854	52.6	
Hispanic	198	7.5	380	21.4	344	12.3	1074	58.9	
Asian	27	2.5	99	19.3	52	5.4	467	72.7	
Other	111	17.8	166	22.1	132	14.4	288	45.7	
Marital status									<.001
Married	533	7.0	1792	26.3	736	7.8	4301	58.9	
Widowed	43	4.0	296	32.1	40	4.0	548	59.9	
Divorced/separated	273	10.0	607	30.2	282	11.1	940	48.4	
Never married	678	13.0	846	19.6	1263	21.3	2349	46.3	
Education									<.001
Less than high school	174	8.9	352	28.4	196	7.4	721	55.3	
High school	403	8.5	847	26.8	590	11.2	1894	53.5	
Some college/associate	580	12.0	1189	26.6	772	12.0	2329	49.9	
College	370	5.5	1153	24.5	763	10.4	3194	59.6	
Poverty level									<.001
<100% FPL	359	13.0	580	29.1	389	11.4	1081	46.8	
100%-200%	361	9.0	719	27.5	493	12.0	1481	51.4	
>200% FPL	806	7.5	2242	25.1	1439	10.4	5573	56.9	
Insurance coverage									<.001
Private insurance	728	7.3	1988	24.3	1410	10.8	5192	57.6	
Public insurance	677	10.0	1398	30.3	722	10.0	2489	49.6	
No insurance	122	12.0	155	17.1	189	17.3	457	53.7	
Metropolitan status									<.001
Large metro	640	8.3	1500	25	1041	11.6	3597	55.1	
Small metro	633	8.5	1393	26.1	941	11.2	3268	54.2	
Non-metro	254	9.1	648	30.2	339	7.6	1273	53.0	
Multimorbidity									<.001
Yes	525	9.4	1471	32.4	468	8.4	2402	49.8	
No	1002	8.0	2070	22.4	1853	12.3	5736	57.3	
Body Mass Index									<.001
Underweight/normal	397	8.1	670	20.3	766	13.4	2231	58.2	
Overweight	426	8.5	971	23.7	618	10.1	2399	57.7	
Obese	679	8.9	1799	30.6	906	10.0	3339	50.5	
Smoking status									<.001
Current smoker	517	21.0	496	24.8	508	17.1	659	37.0	
Former smoker	395	10.0	951	29.4	504	11.3	1695	49.3	
Never smoked	613	4.8	2091	24.9	1304	9.1	5779	61.3	
Alcohol use									<.001
Heavy drinker	154	22.0	105	18	250	24.8	233	35.3	
Moderate	761	9.4	1492	24.5	1376	14.4	3548	51.8	
Did not drink past month	524	8.1	1437	29.6	569	8.3	2776	54.0	
Abstainer	38	1.3	467	25.3	46	1.2	1514	72.2	
Benzodiazepine use within the year									<.001
Yes	428	19.0	619	36.4	265	11.8	522	32.7	
No	861	6.8	2682	25.4	1472	9.5	6661	58.3	
COVID and mental health									<.001
No effect	370	6.1	1205	26.4	524	7.6	2945	59.8	
Little effect	733	8.2	1730	26.4	1191	10.9	4077	54.5	
Quite a bit/a lot	393	16.0	550	24.6	562	19.1	1034	40.4	

Abbreviation: Wt, weighted.

Based on 15 527 adult participants aged 18 years or older with chronic conditions from the 2022 National Survey on Drug Use and Health. Missing categories of poverty status, alcohol use, smoking, and body mass index are not presented. Group differences in marijuana and opioid use categories in the past 12 months were tested with Rao-Scott chi square statistics.

Table 3 presents the sample characteristics by whether the individual had MDD. Overall, 9.3% of individuals with chronic conditions had MDD. MDD prevalence differed significantly (*P* < .001) across opioid and marijuana use categories. Specifically, individuals with co-use of opioids and marijuana had the highest prevalence of MDD (20.8%), followed by individuals with marijuana use only (18.4%), opioid use only (9.3%), and neither substance use (5.6%). MDD prevalence was also significantly associated with demographic and social determinants of health factors. For example, MDD prevalence was significantly higher among women (11.2%) than men (7.1%) and was lower among non-Hispanic Black individuals (7.6%) compared to non-Hispanic White individuals (9.4%).

**Table 3.** Description of Selected Characteristics by Major Depressive Disorder Among Adults (>18 years) with Chronic Conditions, National Survey of Drug Use and Health (NSDUH), 2022.

	Major depression		No major depression		Chi-square	*P*-value
Group	N	Wt%	N	Wt%		
ALL	2042	9.3%	13 485	90.7%		
Marijuana and opioid use past 12 mo					244.285	<.001
Co-use	426	20.8	1101	79.2		
Opioid use	441	9.3	3100	90.7		
Marijuana use	502	18.4	1819	81.6		
Neither	673	5.6	7465	94.4		
Sex					33.799	<.001
Female	1403	11.2	7480	88.8		
Male	639	7.1	6005	92.9		
Age in years					238.438	<.001
18-23	529	26.9	1488	73.1		
24-34	602	19.1	2355	80.9		
35-49	543	11.7	3529	88.3		
50-64	257	8.8	2534	91.2		
65+	111	3.5	3579	96.5		
Race and ethnicity					5.868	.209
Non-Hispanic White	1367	9.4	8887	90.6		
Non-Hispanic Black	183	7.6	1596	92.4		
Hispanic	271	9.0	1725	91.0		
Asian	64	9.3	581	90.7		
Other	157	14.2	696	85.8		
Marital status					139.665	<.001
Married	592	5.9	6770	94.1		
Widowed	52	5.5	875	94.5		
Divorced/separated	308	12.2	1794	87.8		
Never married	1090	16.8	4046	83.2		
Education					12.972	.005
Less than high school	184	8.0	1259	92.0		
High school	509	9.2	3225	90.8		
Associate/some college	772	11.2	4098	88.8		
College	577	7.8	4903	92.2		
Poverty level					34.681	<.001
<100% FPL	446	13	1963	87		
100%-200%	490	12.3	2564	87.7		
>200% FPL	1106	7.7	8954	92.3		
Insurance coverage					24.763	<.001
Private insurance	1096	7.7	8222	92.3		
Public insurance	794	11.3	4492	88.7		
No insurance	152	13.4	771	86.6		
Metropolitan status					5.581	.061
Large metro	785	8.5	5993	91.5		
Small metro	897	10.5	5338	89.5		
Non-metro	360	9.3	2154	90.7		
Multimorbidity					0.942	.332
Yes	654	9.7	4212	90.3		
No	1388	9.0	9273	91.0		
Body Mass Index (BMI)					14.371	.002
Underweight/Normal	575	10.5	3489	89.5		
Overweight	479	7.0	3935	93.0		
Obese	952	10.3	5771	89.7		
Smoking status					32.361	<.001
Current smoker	428	15.3	1752	84.7		
Former smoker	386	8.1	3159	91.9		
Never smoked	1225	8.3	8562	91.7		
Heavy drinking status					28.789	<.001
Heavy drinker	126	11.9	616	88.1		
Moderate	921	9.4	6256	90.6		
Did not drink past month	772	10.4	4534	89.6		
Abstainer	185	4.6	1880	95.4		
Benzodiazepine use within the year					93.282	<.001
Yes	513	19.6	1489	80.4		
No	1529	7.7	11 996	92.3		
COVID and mental health					457.315	<.001
No effect	265	3.9	4779	96.1		
Little effect	886	8.4	6845	91.6		
Quite A Bit/A lot	859	27.5	1680	72.5		

Abbreviation: Wt, weighted.

Based on 15 527 adult participants aged 18 years or older with chronic conditions from the 2022 National Survey on Drug Use and Health. Missing categories on poverty status, alcohol use, smoking, and body mass index are not presented. Group differences in marijuana and opioid use past 12 months were tested with Rao-Scott chi square statistics.

Table 4 presents the results of the multivariable logistic regression analyses examining the association between past-year marijuana and opioid use and MDD. In the unadjusted model (Model 1), individuals who reported co-use of marijuana and opioids in the past 12 months were over 4 times as likely to meet criteria for MDD compared to those who used neither substance (UOR = 4.42; 95% CI: 3.45-5.66). Meanwhile, those who used opioids only (UOR = 1.74; 95% CI: 1.42-2.13) and those who used marijuana only (UOR = 3.81; 95% CI: 3.00-4.84) also had significantly higher odds of MDD compared to non-users.

**Table 4.** Unadjusted and Adjusted Odds Ratios (AOR) and 95% CI of Opioid and Marijuana Use Categories From Logistic Regression on Major Depressive Disorder, Adults (>23 years) with Chronic Conditions National Survey of Drug Use and Health (NSDUH), 2022.

Model and factors	UOR	95% CI	*P*-value
Model 1			
Marijuana & opioid use past 12 mo			
Co-use	4.42	[3.45, 5.66]	<.001
Opioid use	1.74	[1.42, 2.13]	<.001
Marijuana use	3.81	[3.00, 4.84]	<.001
Neither (ref)			
Model 2: Adjusted for sex, age, race and ethnicity			
	AOR	95% CI	*P*-value
Marijuana & opioid use past 12 mo			
Co-use	3.43	[2.76, 4.26]	<.001
Opioid use	1.83	[1.50, 2.24]	<.001
Marijuana use	2.55	[1.97, 3.29]	<.001
Neither (ref)			
Model 3: Adjusted for sex, age, race and ethnicity, marital status, education, poverty status, health insurance, metro status, multimorbidity, body mass index, smoking, and alcohol use			
Marijuana & opioid use past 12 mo			
Co-use	2.62	[2.04, 3.37]	<.001
Opioid use	1.61	[1.30, 2.00]	<.001
Marijuana use	2.10	[1.62, 2.74]	<.001
Neither (ref)			
Model 4: Fully adjusted for sex, age, race and ethnicity, marital status, education, poverty status, health insurance, metro status, multimorbidity, body mass index, smoking, alcohol use, covid-related mental health, and benzodiazepine use in the past year			
Marijuana & opioid use past 12 mo			
Co-use	1.91	[1.45, 2.50]	<.001
Opioid use	1.44	[1.14, 1.82]	.0015
Marijuana use	1.72	[1.31, 2.26]	<.001
Neither (ref)			

Based on 15 527 adult participants aged 18 or older, with chronic conditions from the 2022 National Survey on Drug Use and Health. Missing indicators for poverty status, heavy drinking, heavy smoking and body mass index categories were included in the regression analyses.

After sequentially adjusting for demographic, socioeconomic, and health status (Models 2 and 3), the odds ratios decreased but remained above 1 (*P* < .001), indicating higher adjusted odds of MDD among all 3 user groups compared to adults who did not use either marijuana or opioids. In the fully adjusted model (Model 4), which controlled for additional covariates such as COVID-related mental health concerns, multimorbidity, and benzodiazepine use, co-use remained significantly associated with MDD (AOR = 1.91; 95% CI: 1.45-2.50), indicating 91% higher odds compared to individuals who used neither marijuana nor opioids. Those who used opioids only (AOR = 1.44; 95% CI: 1.14-1.82) and marijuana only (AOR = 1.72; 95% CI: 1.31-2.26) continued to have significantly higher adjusted odds of MDD as well.

## Discussion

This nation-wide study set out to examine the association of co-use of marijuana and opioids with MDD among US adults with chronic conditions. Our study found that 8.5% of adults 18 years or older with chronic conditions reported co-use of marijuana and opioids Previous studies have reported a broad range of co-use rates, from as low as 1% in some populations with persistent pain to as high as 45% in certain subgroups (eg, pregnant women), highlighting the influence of demographic and health status on these patterns. Although pain was not directly measured in the NSDUH, both opioids and marijuana are commonly used to manage pain, and individuals with chronic conditions are particularly likely to use these substances for analgesic purposes.^12,36-41^ Accordingly, our focus on adults with chronic conditions, who are more likely to experience pain, provides an important context in which to understand the potential overlap between pain management behaviors and mental health outcomes.

Our main findings on the association of co-use with MDD consistently demonstrated the largest odds ratios, suggesting that concurrent use of marijuana and opioids carries a distinct, stronger association with MDD than using either substance alone. However, our study focused on adults with chronic conditions and to-date, this is the first study on the association between combined use of marijuana and opioids and MDD among persons with chronic conditions. Substantially greater odds of MDD among those with co-use than the use of either substance alone, suggests a potential synergistic interaction. In a survey of U.S. college students, Lui et al^42^ found that polysubstance users, defined as those reporting use of 2 or more psychoactive substances, had significantly higher odds of both depression and anxiety compared with single-substance users. Similarly, MacDonald et al^43^ examined clients dependent on alcohol, cocaine, or both, and reported that individuals with concurrent alcohol–cocaine dependence scored significantly higher on validated anxiety and depression symptom scales than those dependent solely on cocaine. Taken together, these studies provide convergent evidence that the combined use of multiple substances confers a greater risk for depression than use of any 1 drug in isolation, a potential interactive effect.

Our study also found that use of marijuana and opioids may differentially affect MDD in adults with chronic conditions. Individuals who reported using marijuana use alone or opioids alone had significantly higher odds of MDD compared to non-users, the adjusted odds of marijuana use with MDD was higher. Although the relationship of marijuana and opioids with MDD are complex, individuals who use marijuana may be more susceptible to MDD because marijuana can impact various brain regions involved in mood, cognition, and pain.^44^ This can lead to a complex interplay of effects, including potential for MDD. However, when we compared the odds of MDD among individuals who use marijuana alone (aOR = 1.18, 95% CI = 0.93, 1.49) with opioid use alone, they were not significantly different, a finding that diverges from a study in Israel,^19^ which reported a stronger association of opioid use with depression.

Our findings suggest that addressing co-user patterns may require approaches that go beyond traditional models treating substance use or depression in isolation. There is an urgent need to closely monitor, and screen mental health and substance use among US adults with chronic conditions in mental health as well as in primary care settings. Primary care professionals should be trained to assess poly-substance use patterns, particularly in patients with depressive symptoms, and to tailor treatment strategies accordingly. This monitoring is increasingly important given that more states are relaxing regulations on marijuana, leading to increased marijuana access. Similarly, traditional mental health treatment models that address substance use in isolation may be insufficient for co-users. Co-users may benefit more from holistic programs that integrate psychotherapeutic solution-focused approaches and with addiction-focused strategies such as evidence-based recovery, medication-assisted treatment, residential treatment, motivational interviewing, and cognitive behavioral therapy. Research supports integrated treatment models for comorbid substance use and depressive disorders as more effective than parallel or sequential approaches.^45,46^ Although not the focus of the study, our findings revealed that sociodemographic characteristics, health factors, and COVID-19-related variables can influence both substance-use patterns and the risk of MDD among adults with chronic conditions. For example, differences by age, sex, race and ethnicity may reflect variation in substance use patterns and structural inequities, such as unequal exposure to stress, differences in pain treatment and prescribing, and uneven access to supportive services, that can influence both substance use and mental health.^18,47^ Likewise, socioeconomic characteristics (eg, education, income, and insurance coverage) can also affect access to nonpharmacologic pain management, specialty mental health services, and continuity of care. These factors may decrease reliance on substances while also reducing untreated depressive symptoms.^48,49^ Health-related factors, including the number and type of chronic conditions and self-rated health, may further increase vulnerability to both co-use and MDD through greater symptom burden, functional limitation, sleep problems, and stress associated with living with chronic illness.^50^

In our study those with multimorbidity had notably higher prevalence of both co-use and opioid-only use compared with those without multimorbidity. This pattern is consistent with prior literature indicating that individuals with multiple chronic conditions often experience more persistent or severe pain, leading to increased reliance on pharmacologic pain management approaches.^39,51,52^ The elevated rates of co-use and opioid-only use among adults with multimorbidity in our study underscore the possibility that greater chronic disease burden may contribute to more complex substance-use patterns and, consequently, at higher risk of adverse mental health outcomes such as MDD. These findings highlight the importance of integrated care approaches that substance use, multimorbidity, and mental health simultaneously.

Finally, the COVID-19 period added an extra layer of strain. Disruptions in healthcare, increased isolation, financial instability, and pandemic-related distress may have shifted substance-use behaviors and contributed to a higher risk of depressive episodes.^53,54^ Overall, these findings suggest that co-use and MDD do not occur in isolation and can be affected by patients’ social and health circumstances. Therefore, effective screening and intervention strategies should be implemented.

### Strengths and Limitations

This study has several strengths. MDD, opioid use, and marijuana use were available from a large, long-established, U.S. national study with validated questions. The large sample size permitted the inclusion of many covariates that could be influential in the relationship of MDD and opioid and/or marijuana use. Importantly, our models adjusted for the self-perceived mental health impact of COVID-19, which has been identified as a major population-level stressor contributing to depressive symptoms worldwide. Adjusting for this factor minimized potential confounding from pandemic-related distress that might otherwise bias the associations between opioid and marijuana use and MDD. Nevertheless, the data set for this study did place some limitations upon our analyses. We were not able to distinguish between opioid use by prescription and not directed by a doctor. Further, the NSDUH does not capture detailed information on non-prescribed opioid use, such as opioids obtained outside of medical settings (eg, obtaining street drugs). Data were not available concerning marijuana use by prescription, or self-use in states where allowed. Co-use may not represent use of both substances simultaneously. We also did not have data concerning whether the substances were being used for pain relief or for other purposes. Finally, this study identifies a correlation and not a causal relation from co-use to MDD: it is possible that MDD causes co-use or that some factors drive both co-use and MDD simultaneously. Because this study analyzed the full adult sample available in the 2022 National Survey of Drug Use and Health (NSDUH), a large nationally representative dataset, no a priori sample size calculation or power analysis was performed.

## Conclusion

One in 12 US adults with chronic conditions (weighted n = 9.28 million) had co-use of marijuana and opioids. Co-use of marijuana and opioids carries a distinct, stronger association with MDD than using either substance alone. There is a need to closely monitor substance use, in particular co-use among US adults with chronic conditions, as early detecting may reduce adverse mental health outcomes.

## Appendix

**Table 1.** Weighted Percent of Chronic Conditions by Overall and Analytical Sample Adults (Age 18 Years or Older), National Survey of Drug Use and Health, 2022.

Group	Adults with chronic conditions (Wt N = 109 066 757)	Adults with and without chronic conditions (Wt N = 250 655 094)
N	15 527	45 874
	Wt%	Wt%
Asthma		
Asthma	24.0	10.4
No asthma	76.0	89.6
Cancer		
Cancer	15.5	6.7
No cancer	84.5	93.3
Cirrhosis		
Cirrhosis	1.2	0.5
No cirrhosis	98.8	99.5
COPD		
COPD	9.3	4.0
No COPD	90.7	96.0
Kidney disease		
Kidney disease	5.4	2.4
No	94.6	97.6
Diabetes		
Diabetes	27.2	11.8
No diabetes	72.8	88.2
Heart disease		
Heart disease	25.3	11.0
No heart disease	74.7	89.0
Hypertension		
Hypertension	46.9	20.4
No hypertension	53.1	79.6
Hepatitis		
Hepatitis B/C	2.6	1.1
No Hepatitis B/C	97.4	98.9
HIV/AIDS		
HIV/AIDS	1.0	0.4
No HIV/AIDS	99.0	99.6

Based on adult participants aged 18 or older, with (N = 15 527) and without (N = 45 874) chronic conditions from the 2022 National Survey on Drug Use and Health.
